# Dr. George H. Chance

**Published:** 1904-11

**Authors:** 


### Obituary.

### DR. GEORGE H. CHANCE.

The announcement of the death of Dr. Chance, of Portland, Ore., will create a feeling of serious loss to the dental profession throughout the country, but this will be more pronounced upon the Pacific coast where his long and active service has made his name familiar to all in dental practice. He has demonstrated in his life and work the very best of that type of the pioneer dentists of the western coast, a type honored throughout the United States for the high standard maintained in education and practice.

The personal intercourse of the writer with Dr. Chance was necessarily limited, but it was sufficient to mark him as a man genial in disposition, lovable in character, and earnest always for the best in his profession.

One by one the old standard-bearers are passing to the eternal beyond. This demands at our hands a more serious devotion to the work they left uncompleted. We must emulate, and surpass if possible, their unselfish devotion to a great trust. They went up to the altar of sacrifice and carried there the best fruits of their lives. Let us aim to follow in their footsteps.

Dr. Chance worked to almost the last hour of active life. He was taken sick while attending the California State Society. Up to the time of this visit he had been in active practice. His sufferings from that hour were severe. An operation enabled his friends to remove him to his home, where he lingered for over two months.

Dr. Chance was born in England seventy-four years ago, and came to this country about the year 1850. The gold regions of California drew him to the Pacific coast, and about 1860 he settled in Salem and lived there until 1874 when he removed to Portland, where he lived until the time of his death.

Dr. Chance's last visit East, it is believed, was in attendance at the National meetings held at Niagara Falls a few years since. He accompanied the National Association of Faculties upon a trip to Toronto, and those who were with him on that trip will not soon forget the pleasure he gave by his conversation and thorough enjoyment manifested in association with his professional co-workers. How thoroughly he enjoyed life is manifest by this quotation from his little book entitled "Sunshine Thoughts for Gloomy Hours."

"Is it not better for us to take a broader and therefore a clearer view of this life than most of us do, and soar for awhile above the clouds and thus get a little more sunshine into our souls. On our journey to what we all hope will prove to be the larger and the better life, we pass this way but once; let us keep our eyes for the bright spots in the landscape, for the fruits, the birds, and the flowers, and avoid, in all possible ways, the poisonous swamps of pessimism."

As a dentist Dr. Chance enjoyed a large practice. He was an active official in the Taylor Street Methodist Episcopal Church. He was also a thirty-third degree Mason.

He leaves a widow and the following children: Mrs. F. A. Kenny, Alameda, Cal.; Miss S. E. Chance, Miss W. E. Chance, Miss Alna B. Chance, Charles H. Chance, and Dr. Arthur W. Chance.

The funeral took place September 5, 1904.

